# Association of lymphocyte to monocyte ratio with severity of coronary artery disease

**DOI:** 10.1097/MD.0000000000012813

**Published:** 2018-10-26

**Authors:** Shu Gong, Ximei Gao, Fubiao Xu, Zhi Shang, Shuai Li, Wenqiang Chen, Jianmin Yang, Jifu Li

**Affiliations:** a The Key Laboratory of Cardiovascular Remodeling and Function Research, Chinese Ministry of Education and Chinese Ministry of Public Health; b International Medical Department, Qilu Hospital, Shandong University, Jinan; c Department of Cardiology, Heze Municipal Hospital, Heze, Shandong, China.

**Keywords:** atherosclerosis, coronary artery disease, Gensini score, lymphocyte to monocyte ratio

## Abstract

The aim of this study was to investigate the relationship between lymphocyte to monocyte ratio (LMR) and the severity of coronary artery disease (CAD) by using Gensini score.

A total of 199 patients, who had undergone coronary angiography, were included in the study and retrospectively analyzed. Among them, 49 patients who had normal coronary arteries were selected as the control group. Patients with CAD were divided into 2 groups, those with low Gensini score (≤40) and those with high Gensini score (≥40).

Our results showed that LMR in the severe atherosclerosis group was significantly lower than those of the mild atherosclerosis group and the control group. There was a closely significant correlation between the Gensini score and LMR (*r* = −0.362, *P* < .001). Furthermore, multivariate logistic regression analysis demonstrated that LMR (odds ratio, 0.715; 95% confidence interval [CI], 0.551–0.927; *P* = .012) was independent predictors of severe atherosclerosis. Using an optimal LMR cut-off value of 5.06, LMR predicted severe atherosclerosis with a sensitivity of 57.1% and specificity of 69.7% (area under curve = 0.634; 95% CI, 0.545–0.724; *P* = .005). Then patients with CAD group was divided into 2 groups according to the LMR value of 5.06. Patients with LMR ≤ 5.06 had worse prognosis, with a higher rate of cardiovascular events during up to 1 year follow-up.

Our study demonstrated that LMR was independently and positively associated with the severity of coronary atherosclerosis, providing a new insight in the application of inflammation index evaluating the severity of CAD. And LMR may be a useful predictor of future cardiovascular events in patients with CAD.

## 1 Introduction

Recently, coronary artery disease (CAD) remains the most common cause of morbidity and mortality in the general population. Chronic inflammation is involved in all stages of coronary atherosclerosis, from initial endothelial dysfunction and plaque disruption to clinical manifestation of acute atherothrombotic events.^[1]^ Among the various inflammatory markers, the leukocyte count and whole subtypes of white blood cell counts including neutrophils, monocytes, and lymphocytes are associated with increasing cardiovascular events.^[2,3]^ Earlier studies have demonstrated that lymphocyte count is inversely correlated with inflammation and lower lymphocyte count represented increased cardiovascular risk and mortality.^[4,5]^ Previous study showed that neutrophil to lymphocyte ratios (NLRs) were emerging markers of the incidence and severity of CAD.^[6,7]^ Furthermore, lower lymphocyte counts and higher monocyte counts were associated with adverse cardiovascular endpoints in patients with CAD.^[8,9]^ Therefore, a composite maker of inflammation reflecting low lymphocyte and high monocyte may provide additive information in the assessment of cardiovascular risk. Recently, the lymphocyte to monocyte ratio (LMR) has been found to be a novel systemic inflammatory marker. With respect to the vascular diseases, it has revealed that a decreased LMR was responsible for the adverse outcomes in peripheral arterial occlusive diseases.^[10]^ However, clinical studies focused on assessment the relationship between LMR and severity and prognosis of CAD in patients was relatively rare and more researches remain to be done.

The LMRs, which is inexpensive, commonly used, reproducible, and widely available in clinical practices, have been proven to be an important inflammatory marker and potential predictor of cardiovascular atherosclerosis risk. It is widely accepted that inflammatory reactions play a vital part in the pathogenesis of atherosclerosis, which offer us new inspirations for the prevention and treatment of CADs. Thus, the aim of our study was to assess the association of LMR and severity of CAD.

## 2 Methods

A total of 199 consecutive patients with known or suspected CAD undergoing diagnostic coronary angiography at Shandong University Qilu Hospital between June 2015 and November 2016 were enrolled in this study. All patients have complete information. Exclusion criteria included recent acute coronary syndrome, history of previous coronary intervention or coronary artery bypass graft, severe valvular heart disease, decompensated heart failure, cerebrovascular disease, renal or hepatic disease, acute or chronic infection and/or inflammation, malignancy, malnutrition, hematologic disease, symptomatic peripheral arterial disease, autoimmune disease, pregnancy, and chronic obstructive lung disease. Arterial hypertension was defined as a systolic blood pressure ≥140 mm Hg and/or a diastolic blood pressure ≥90 mm Hg in at least 2 measurements or a history of antihypertensive drugs. Diabetes mellitus was defined as fasting plasma glucose ≥126 mg/dL at any measurements or having a glycated hemoglobin fraction of ≥6.5% or a history of antidiabetic drug. Smoking status was defined as current smoker or nonsmoker. Patients who had quit smoking >1 month prior to admission were classed as nonsmoker. Informed consent was obtained from all included patients before coronary intervention, and the study protocol was approved by the local ethics committee.

### 2.1 Baseline variables

In our hospital, blood samples are collected from the ante-cubital vein within 24 hours of hospital admission. Venous blood samples were taken into vacutainer tubes (using sodium heparin as anticoagulant). Complete blood counts, which included the total white blood cells, hemoglobin, platelets, neutrophils, lymphocytes, and monocytes, were all measured with an auto-analyzer. Plasma levels of total cholesterol, triglycerides, high-density lipoprotein cholesterol, low-density lipoprotein cholesterol, creatinine and glucose were evaluated using an automated chemistry analyzer. All patients were assessed with transthoracic echocardiography. The left ventricular ejection fraction (LVEF) was calculated using the Modified Simpson method. LMR was calculated by dividing the lymphocyte count with the monocyte count.

### 2.2 Coronary angiography and Gensini score

Coronary angiography was performed using standard Judkins technique. At least 2 different plane images were displayed for each coronary artery. CAD was defined as the presence of obstructive stenosis of >50% of the vessel lumen diameter in any of the main coronary arteries, including the left main coronary artery, left anterior descending (LAD) artery, left circumflex coronary artery and right coronary artery, or main branches of the vascular system. The Gensini score system was used in evaluating the severity of the CAD. In this system, 1 point is given for ≤25% lumen stenosis, 2 points for 26% to 50% lumen stenosis, 4 points for 51% to 75% lumen stenosis, 8 points for 76% to 90% lumen stenosis, 16 points for 91% to 99% lumen stenosis, 32 points for total occlusion. This score is multiplied by coefficients that demonstrated the importance of the lesion's position in the coronary circulation, such as 5 for left main coronary artery, 2.5 for proximal LAD and proximal left circumflex (LCX), 1.5 for the mid-segment LAD, 1 for the distal segment of LAD and LCX, the mid or distal first diagonal branch, first obtuse marginal branch, right coronary artery, posterior descending artery and intermediate arteries, and 0.5 for other segments. The total score equals each luminal stenosis and the coefficient. The mild atherosclerosis group (n = 66) was defined for Gensini score between 1 and 40, and the severe atherosclerosis group (n = 84) was defined for Gensini score ≥40.^[11]^ All coronary angiograms were evaluated by 2 experienced interventional cardiologists.

### 2.3 Follow-up and cardiovascular events

The composite rate of adverse CVD events, including cardiac related death, myocardial infarction [MI], and stroke, was obtained by telephone interviews at 1 year after coronary angiography. Cardiac death was defined as death resulting from an evident cardiac cause or any death related to PCI. Nonfatal MI was defined as the recurrence of chest pain and/or the development of new ECG changes accompanied by a new rise ≥20% of cardiac biomarkers measured during the event. Stroke was defined as an acute neurologic deficit consistent with recent ischemic or hemorrhagic events.

### 2.4 Statistical analysis

All analyses were conducted using the SPSS 19.0 (SPSS, Chicago, IL). Continuous data were defined as mean ± standard deviation, whereas categorical date is expressed as percentages. Continuous variables were compared with an independent sample *t* test, and categorical variables were compared with Chi-squared test. Correlation analysis between baseline variables and Gensini score was performed by using the Pearson (for parametric variables) and Spearman (for nonparametric variables) rank correlation coefficient. All variables was analyzed by univariate logistic regression analysis, then variable <0.05 in univariate analysis were included in the multivariate logistic regression analysis. Receiver operation characteristic (ROC) curves were used to derive sensitivity and specificity of LMR to predict the presence of severe CAD. Kaplan–Meier analysis was performed in patients with CAD and comparisons of the incidence of cardiovascular events were performed using the log-rank test.

## 3 Results

### 3.1 Baseline characteristics of control and CAD group

The study population was categorized into 3 groups according to coronary angiography including control group with normal coronary arteries (control group, n = 49 patients), study group with CAD (mild atherosclerosis, n = 66 patients), and (severe atherosclerosis, n = 84 patients). There was no statistically significant difference between control group and study group by means of age, total cholesterol, triglycerides, low-density lipoprotein cholesterol, creatinine, glucose, LVEF, hemoglobin, platelet, lymphocyte, and the presence of diabetes mellitus. No statistically significant difference was found in prior treatment between control group and study group. The rate of male gender, hypertension, smoking, white blood cell, neutrophil, and monocytes were higher in study group as compared with control group (*P* < .05). However, high-density lipoprotein and LMR were significantly lower in the CAD group compared with the control group (Table 1).

**Table 1:**
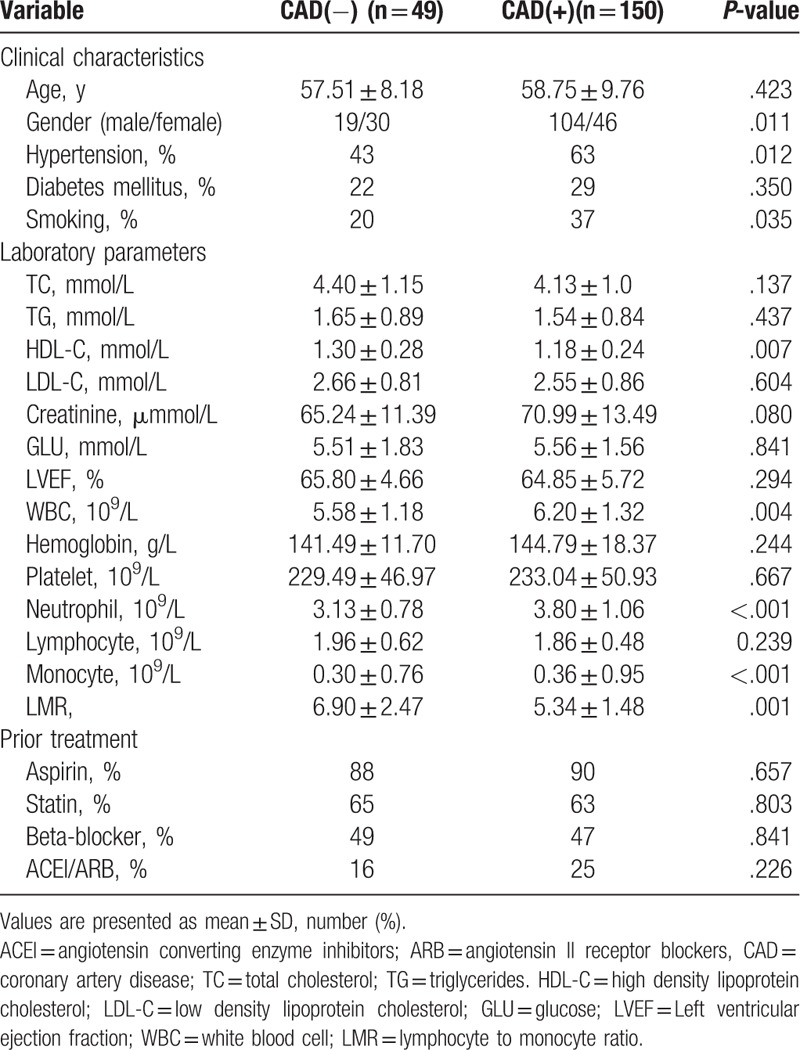
Baseline characteristics of Control group and CAD group.

### 3.2 Baseline characteristics of mild atherosclerosis and severe atherosclerosis

Those in the severe atherosclerosis group had significantly higher glucose, whereas the group that had lower Gensini score tended to have higher LVEF, high-density lipoprotein cholesterol, lymphocyte, and LMR (Table 2).

**Table 2:**
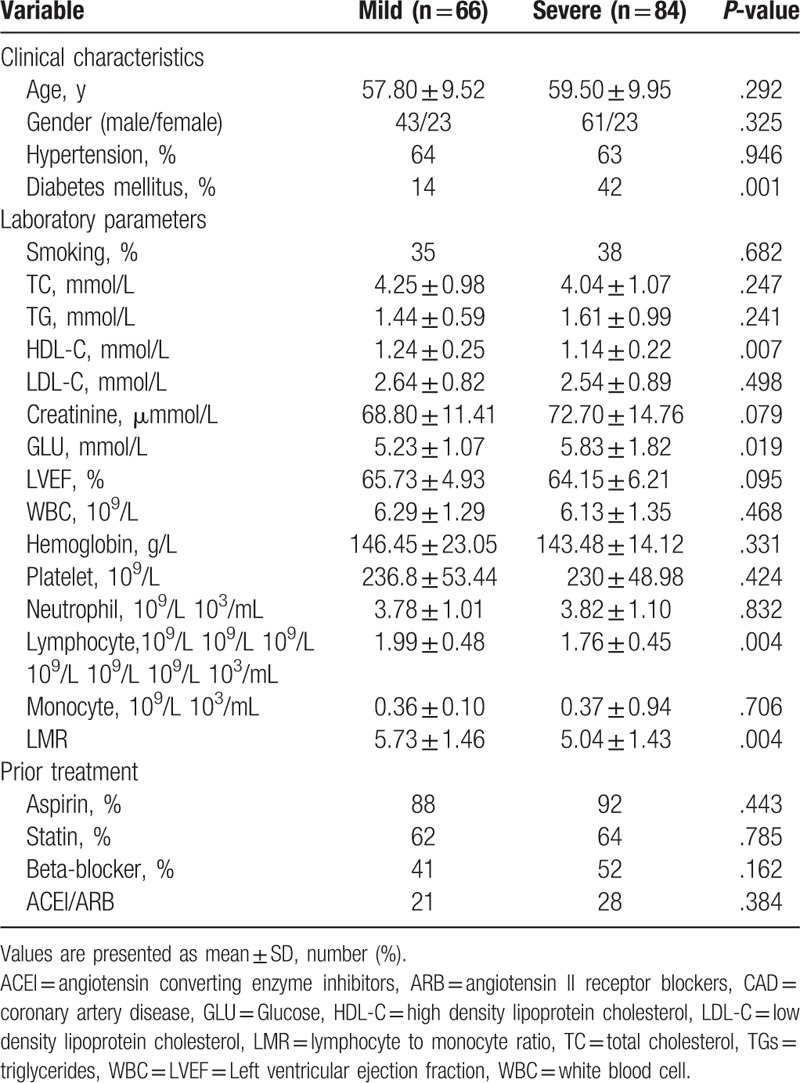
Baseline characteristics of mild and severe atherosclerosis.

### 3.3 Correlation of baseline characteristics and Gensini score

In the correlation analysis, Gensini score was significantly positively correlated with the rate of male gender, diabetes mellitus, creatinine, neutrophil, and monocytes, it was significantly negatively correlated with high-density lipoprotein, LEVF, lymphocyte and LMR (Table 3).

**Table 3:**
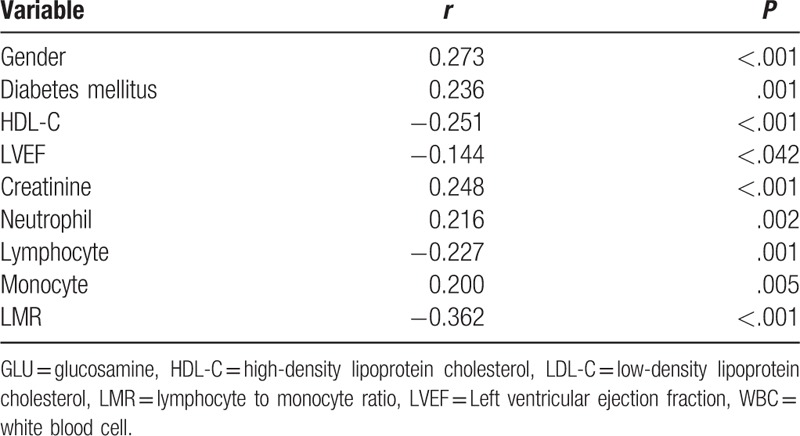
Correlation of baseline characteristics and Gensini score.

### 3.4 Predictors of the severe coronary atherosclerosis by logistic regression analysis

The statistically significant variables in the correlation analysis were assessed by logistic regression analysis. On univariate analysis, the predictors of severe coronary atherosclerosis were diabetes mellitus, high-density lipoprotein cholesterol, and LMR (*P* < .05). In multivariate logistic regression analysis, the independent predictors of the severe CAD were diabetes mellitus (odds ratio [OR], 4.909; 95% confidence interval [CI], 2.056–11.722; *P* < .001), high-density lipoprotein (OR, 0.205; 95% CI, 0.044–10.968; *P* = .045), and LMR (OR, 0.715; 95% CI, 0.551–10.927; *P* = .012) (Table 4).

**Table 4:**
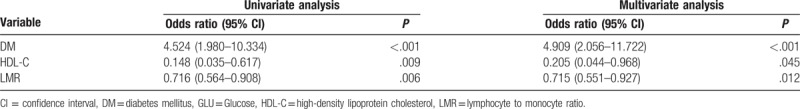
Univariate and mulitivariate logistic regression analysis showing the predictors of the severe coronary atherosclerosis.

### 3.5 Receiver-operating characteristic analysis of lymphocyte, monocyte and LMR for severe atherosclerosis

**Figure 1:**
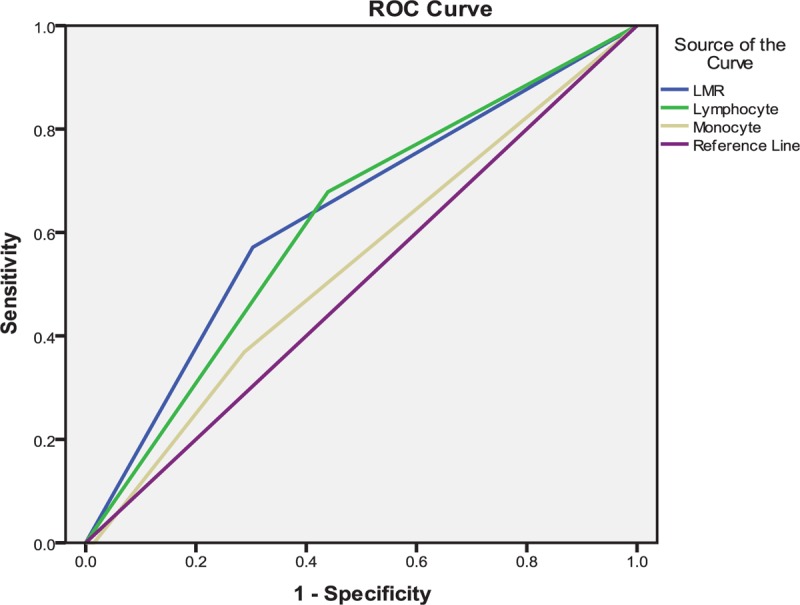
Diagnostic accuracy of lymphocyte, monocyte, and lymphocyte to monocyte ratio (LMR) in prediction of coronary artery disease.

In the ROC curve analysis, monocyte did not significantly predict severe atherosclerosis. However, it was revealed that using a cut-off level of 1.91, lymphocyte predicted severe atherosclerosis with a sensitivity of 67.9% and specificity of 57.1% (area under curve [AUC] = 0.620). In addition, LMR was found to have the area under the curve (AUC = 0.634, *P* = .005) with an optimal LMR cut-off value of 5.06 (sensitivity 57.1%, specificity 69.7) (Fig. 1).

### 3.6 Association of LMR with cardiovascular events in patients with CAD

**Figure 2:**
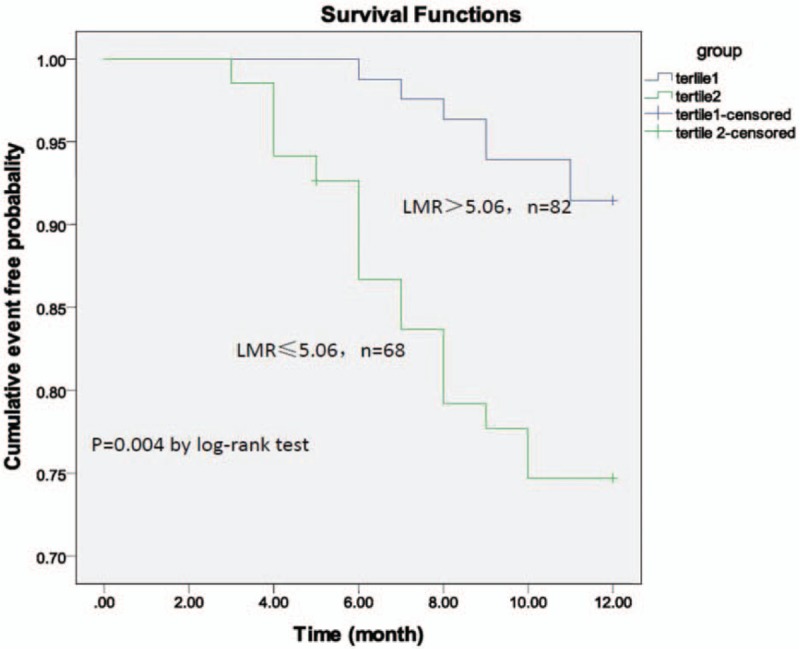
The Kaplan–Meier analysis shows the cumulative event-free composite rate of death, nonfatal myocardial infarction (MI) and stoke according to the optimal cut-off value of a LMR of 5.06.

The patients were stratified into Tertile 1 (LMR > 5.06, n = 82) and Tertile 2 (LMR ≤ 5.06, n = 68) according to the LMR value of 5.06. Cardiac death and nonfatal MI were significantly lower in patients with a high LMR compared to the patients with a low LMR. There were no significant differences in stoke among the groups (Table 5). Kaplan–Meier analysis revealed a higher risk for future cardiovascular events in patients with CAD with a low LMR (*P* = .004 by log-rank test, Fig. 2).

**Table 5:**
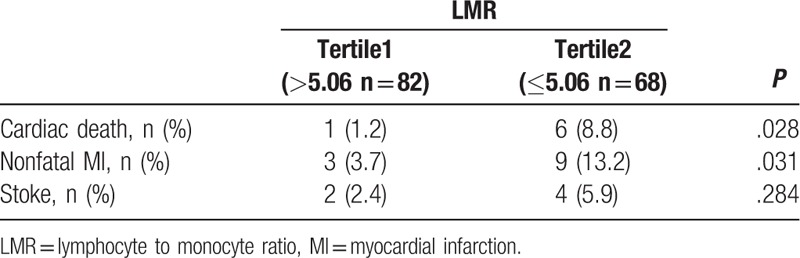
cardiovascular events of the study population according to LMR.

## 4 Discussion

In the present study, we collected 199 patients and retrospectively analyzed the correlation between subtypes of white blood cell and the severity of CAD. To the best of our knowledge, our study revealed that a significant correlation between LMR and severity of CAD. Moreover, it also provided that LMR is an independent predictor of severity of human CAD.

Inflammation plays an active role both in the initiation and progression of atherosclerotic process.^[12]^ However, it is unclear that which cell is the primary responsible for initiation of this cascade processes. The leukocyte, as a marker of inflammation, is widely available in clinical practice. Several previous studies have evaluated the association between the white cell and its subtypes and presence and prognosis of CAD. Lymphocyte plays a crucial role in modulating the inflammatory response in atherosclerotic process.^[13]^ Lymphocyte is involved in the regulatory pathway of the immune system^[14]^ and inflammation increases lymphocyte apoptosis.^[15]^ Transendothelial migration of leukocytes is a key step in the formation of atherosclerosis; they provide human genetic support for a role of this pathway in CAD.^[16]^ Low lymphocyte count reflected the suppressed immune response, which has been shown to be associated with worse clinical outcomes in various cardiovascular diseases.^[15]^ Besides, previous studies have found that low lymphocyte count has been significantly related to untoward events in patients with CAD.^[5,15]^ NLR was calculated by dividing the neutrophil count with the lymphocyte count. The NLR was a combination of 2 independent markers of inflammation. Some studies found that the association of NLR with various cardiovascular diseases and its possibility of emerging as a cheap, reliable, and independent prognostic marker of cardiovascular disease. First, previous studies found that NLR, independently and in interaction with other disease markers and risk factors, is a significant predictor of mortality in stable CAD, as well as its development and progression.^[17,18]^ Then, NLR is an independent predictor of short-term and long-term mortalities in patients with ACS.^[15,18,19]^ A review confirmed that NLR is a significant marker of frequent decompensation and long-term mortality in patients with chronic heart failure, and it also can be used as a prognostic marker for postoperative atrial fibrillation and coronary artery bypass grafting.^[18]^ Atherosclerosis is now regarded as a chronic inflammatory disease, with monocytes and monocyte-derived macrophages playing pivotal roles in coronary plaque progression.^[20–22]^ The recruitment of monocytes into the arterial wall and their development into macrophages are the earliest events in atherosclerosis, indicating the importance of inflammation in vascular endothelial dysfunction and initiation of atherosclerosis. The monocyte count is the most useful marker for the evaluation of atherosclerosis, and is involved at all stages of the progression of atherosclerosis. The article by Yamamoto et al concluded that, of leukocyte differentials, elevated monocyte count independently predicted mid-term cardiovascular events in patients with stable CAD.^[23]^ The monocyte count has also been reported to be an independent predictor of common carotid atherosclerosis in healthy subjects.^[24,25]^ Like NLR, LMR can also act as an inflammatory complex. The LMR is a combination of 2 independent markers of inflammation, and is a novel systemic inflammatory marker. Recently, studies have shown that it is an indicator of systemic inflammatory response and it has been shown to present a potential prognostic factor in various types of malignancies.^[26]^ With respect to the vascular diseases, Gary et al have revealed that a decreased LMR is associated significantly with a high risk for critical limb ischemia and other vascular endpoints in peripheral arterial occlusive disease patients.^[10]^ Recently, research showed the association of LMR with no-reflow phenomenon and in-hospital adverse outcomes in patients who underwent a primary percutaneous coronary intervention for ST-elevationMI.^[27]^ In another recent study, the relationship between LMR and bare-metal in-stent restenosis in patients with SCAD was reported.^[28]^ This study supports that LMR, as an inflammatory biomarker, contributes to the development of atherosclerosis from initiation through progression. More recently, Ji et al found that monocyte to lymphocyte ratio predicts the severity of CAD by a syntax score assessment.^[29]^ However, this study did not exclude the patients with acute coronary event, which might disturb the analysis because such patients may undergo a stress state and acute inflammatory reaction. In our present study, we found that LMRs were significantly lower in the CAD group compared with the control group. Further analysis showed that LMR was significantly correlated with CAD severity. In addition, our study suggested that a cut-off value of LMR 5.06 can predict the presence of atherosclerosis before coronary angiography with a sensitivity of 57.1% and specificity of 69.7%.

Several limitations of this study should be detailed. First, the assessment of the severity of CAD was performed by coronary angiography. Intravascular ultrasound or dual-source multi-slice computed tomographic coronary angiography may be more sensitive in the assessment of the severity of CAD. Second, the study population was small, and an increased number of patients are needed. Additionally, other inflammatory cytokines, such as tumor necrosis-alpha and C reactive protein were not measured. Further multicenter, large-size, prospective study may strengthen our conclusion.

In conclusion, our present study revealed that LMR was significantly correlated with the severity of coronary atherosclerosis in patients with CAD by Gensini score. LMR may be a potential available, easy calculable, and low priced parameter for human coronary atherosclerosis severity prognosis before coronary interventions.

## Author contributions

**Conceptualization:** Jianmin Yang.

**Data curation:** Zhi Shang, Shuai Li.

**Formal analysis:** Jianmin Yang.

**Investigation:** Ximei Gao.

**Methodology:** Fubiao Xu.

**Resources:** Jifu Li, Fubiao Xu.

**Software:** Fubiao Xu.

**Supervision:** Jifu Li, Jianmin Yang, Wenqiang Chen.

**Validation:** Wenqiang Chen.

**Visualization:** Wenqiang Chen.

**Writing – original draft:** Shu Gong, Ximei Gao.

**Writing – review & editing:** Shu Gong, Ximei Gao.
